# TIE: An Ability Test of Emotional Intelligence

**DOI:** 10.1371/journal.pone.0103484

**Published:** 2014-07-29

**Authors:** Magdalena Śmieja, Jarosław Orzechowski, Maciej S. Stolarski

**Affiliations:** 1 Institute of Psychology, Jagiellonian University, Kraków, Poland; 2 Department of Psychology, University of Social Sciences and Humanities, Warsaw, Poland; 3 Faculty of Psychology, University of Warsaw, Warsaw, Poland; French National Centre for Scientific Research, France

## Abstract

The Test of Emotional Intelligence (TIE) is a new ability scale based on a theoretical model that defines emotional intelligence as a set of skills responsible for the processing of emotion-relevant information. Participants are provided with descriptions of emotional problems, and asked to indicate which emotion is most probable in a given situation, or to suggest the most appropriate action. Scoring is based on the judgments of experts: professional psychotherapists, trainers, and HR specialists. The validation study showed that the TIE is a reliable and valid test, suitable for both scientific research and individual assessment. Its internal consistency measures were as high as .88. In line with theoretical model of emotional intelligence, the results of the TIE shared about 10% of common variance with a general intelligence test, and were independent of major personality dimensions.

## Introduction

### Emotional intelligence and its measurement

The concept of emotional intelligence (EI) entered psychological vocabulary more than 20 years ago [1] and quickly earned a high rank of popularity among researchers and practitioners. The original definition conceptualized it as “the ability to monitor one’s own and others’ feelings and emotions, to discriminate among them, and to use this information to guide one’s thinking and actions” ([1], p. 189). Because scientists assumed that this set of abilities explain important life outcomes [2]–[5], they immediately began inventing various psychometric tools suitable for the measurement of EI as an individual trait. With time, the domain split in two assessment strategies. The first relies on self-report questionnaires, and the assumption that people know how well they understand and deal with emotions. According to this approach, EI is typically measured through self-judgments, using items of the form “I often find it difficult to show my affection to those close to me” or “I understand my emotions well.” Self-report questionnaires require thorough insight into one’s mental state based on accurate feedback regarding the accuracy of the emotional abilities, which seems not to be true in most cases [6]. The second assessment strategy uses ability tests based on performance criteria. They are supposed to reflect a person’s actual level of EI development. Although the tests seem much more objective and informative than questionnaires, they require more time and effort, both in the process of their construction and during administration. Another difficulty with the ability tests of EI pertains to the scoring criteria. In the realm of human emotions, problems rarely have only one “true” or “correct” solution; therefore, scoring procedures must be based either on the consensual approach (i.e., the most frequent answer is regarded “correct”) or on painstaking experts’ judgments [7], [8].

The aim of this paper is to introduce a new instrument for assessing emotional intelligence, labeled the Test of Emotional Intelligence (*Test Inteligencji Emocjonalnej*, TIE). Our test is based on the theory developed by Peter Salovey and John Mayer [1], [2], [7]. According to this theory, EI involves a set of cognitive abilities used for processing emotionally relevant information. Specifically, the theory splits EI into four distinct, albeit correlated, abilities, also called “branches”: (a) Perception of Emotions, (b) Using Emotions to Facilitate Thinking, (c) Understanding Emotions, and (d) Managing Emotions [2], [7]. The first ability (Perception of Emotions) consists of proper perception, identification, and recognition of emotions in one’s own subjective experience, as well as in other people’s behavior. The second dimension, labeled Using Emotions to Facilitate Thinking, pertains to the assimilation of emotions with thinking and problem solving. The third ability (Understanding Emotions) consists of proper understanding of emotions, including the comprehension of triggering factors, phases of the emotional process, and proper sequencing of emotional states. Finally, the management of emotions (Managing Emotions) is the ability to regulate one’s own emotional states, as well as the ability to deal with other people’s emotions and feelings.

### TIE: objectives and assumptions

A number of authors argue that EI is important for social functioning and can predict educational achievement or job performance beyond intellectual ability and personality factors [3], [9]–[13]. To explore these relationships, researchers and practitioners need psychometrically sound and conceptually comprehensive measures of EI. Although numerous methods of measurement have already been created [14]–[18], each of the existing tools has some disadvantages. The shortcomings of these measures are different; for some tests it is their length, for others – their insufficient reliability, or the additional equipment needed. Even the Mayer–Salovey–Caruso Emotional Intelligence Test (MSCEIT) [16], which seems the best validated performance measure to fully reflect the ability model of EI, cannot be applied without doubts, as it is not free from cultural specificity [19]. In general, the majority of all concise measures of EI are self-reports influenced by the respondents’ self-esteem and mood, and reflecting rather generalized social adaptation [20] than actual emotional skills, while the ability tests either require considerable time to administer or have limited conceptual coverage. Seeing that a perfect measure of EI has not been created so far, we believe that developing new tools is worthwhile, or even necessary. Both for research and diagnostic purposes, the collection of available tests should be systematically enriched and restored. Since culture can shape the experience and expression of emotions [21], alternative measurement instruments should be developed and validated within different cultural groups. Consequently, our aim was to develop a valid and reliable instrument tapping multidimensional construct of EI, based on narratives and experiences from an adequate cultural context. Staying within scientific bounds in the use of such terms as *emotion* and *intelligence* we opt for the ability-based approach and assume that maximum performance tests are the best tools for assessing EI. Hence, we created a performance-based scale covering the whole set of emotional abilities, which is brief and easy to administer in individual and group settings.

Three assumptions have been made during the construction process of the TIE. First, we wanted to measure the actual abilities of people rather than their own opinions about themselves. The majority of people think that their own EI level is higher than the average [9], therefore performance tests seem much more objective and valid than self-report questionnaires. Second, we intended to create an ecologically valid test, in which solutions would not be scored on the “zero-one” basis. Social and emotional problems are usually too complex and ambiguous to justify such an approach. Therefore, we deliberately decided to give participants the opportunity to rank response options according to their decreasing appropriateness rather than to classify them as “accurate” or “inaccurate”. Third, we decided to base the scoring criteria on experts’ judgment rather than on statistical distribution of the results in the population. Assuming that EI is an ability, or a set of abilities, rather than a personality trait or preference [22]–[24], the most frequent test responses must be regarded as less appropriate than ones that are infrequent but produced by highly emotionally intelligent persons.

### Aims of the study and research hypotheses

The aim of our empirical investigations was threefold. First, we intended to provide a detailed, in-depth look into the factorial structure of EI as measured with the TIE. In addition to the factor analyses, we planned to provide empirical data on TIE reliability and validity. To obtain this purpose, we assessed the test’s internal consistency and reported its relationships with established psychological measures, presenting both its convergent and divergent validity. Because ability tests of EI usually reveal systematic gender and age differences we also analyzed such group differences. Since the TIE is an ability measure of EI, in all cases we expected the results usually obtained for such methods. Below, we present the hypotheses in detail.

#### Factor structure of the TIE

As the test has been based directly on Mayer and Salovey’s four-factor model [2], we first decided to test the factorial solutions endorsed by these authors for their MSCEIT [16], i.e., one-, two- and four oblique-factor solutions. These solutions reflect the authors’ theoretical assumption that general EI (one-factor solution) consists of four branches (four-factor solution), that may be grouped in sets of two due to their functional similarity (two-factor solution): Experiential (perceiving and using emotions) and Strategic (understanding and managing emotions). Additionally, based on initial theoretical analyses and preliminary CFAs conducted for the TIE [25], we propose and test an alternative area division with the abilities of perceiving and understanding emotions constituting the alternative Experiential area, and the abilities of using and managing comprising the new Strategic area.

More recent analyses of the MSCEIT (which remains our main standard of comparison, due to its reputation, popularity, and being the only instrument fully reflecting the ability model of EI) showed that alternative factorial solutions could be more adequate. Therefore, we added a nested modeling procedure endorsed by Palmer and colleagues [26]. This approach does not simply assume that some higher-order factors emerge from the lower-level sets of abilities. Instead, in line with the traditional model of intelligence introduced by Charles Spearman [27], it suggests that test item performance (and, in consequence, life-situation performance) is “loaded” by two different factors: general (the famous “g” factor in intelligence) and specific for particular task/situation (“s” factors). Interestingly, Palmer and colleagues found that EI can also be recognized as a sort of “traditionally understood” intelligence, with one general EI factor, and specific branch-level factors. This observation led us to verify the “nested” (as Palmer calls them) models, to resolve this theoretical bifurcation. Summing up, we decided to test four oblique-factor models: one-factor, four-factor, and two versions of two-factor solutions (with an alternative distribution of subscales between the area-level factors). Further, we applied the “nested” strategy, again testing two versions of two-factor models, a four-factor model, and, additionally, a three-factor model endorsed by Palmer and colleagues [26].

#### General Intelligence

If emotional intelligence indeed represents a kind of intelligence, tests of general mental ability should correlate with tests of EI; however, such correlation should not be very strong in order to exclude the possibility that both domains are impossible to discriminate [7]. Along with this assumption, numerous previous studies have demonstrated that EI, as measured by the ability tests, correlates at a very modest level (from .31 to .39) with verbal intelligence [7], [12], [28]–[30]. In the case of MSCEIT, most of the overlap with verbal intelligence is accounted for by the subscale of Understanding Emotions. Correlations with fluid intelligence are smaller but still significant [12], [31], [32]. We predict analogical results for the TIE.

#### Personality

The results concerning relationships between emotional intelligence and personality are ambiguous, mostly due to the fact that different EI measurement strategies produce different results. Conceptualizing emotional intelligence as a trait and assessing it with self-reports leads to considerable overlap between EI and the Big Five traits [33]–[35]. Not surprisingly, self-judgments of a trait described as a constellation of emotional self-perceptions [36] involving adaptability, assertiveness, social competence, and stress management [37] highly correlates with personality dimensions. Self-judgment scales assess variables relevant to motivation, social skills, and other areas of personality [12], and thus, overlap with the Big Five sometimes as much as the different scales of the Big Five overlap with each other [22]. However, if we define EI as an ability, not a preference or inclination, we should not expect significant relationships with major personality dimensions. Whether or not people are sociable or assertive, they might be intelligent about emotions. Along with that claim, many empirical studies [9], [12], [28], [30] show that ability-based EI shares only a small fraction of common variance with personality, if at all. For example, the MSCEIT correlated .25 with Openness and .28 with Agreeableness [12]. In line with these results, we predict that among five personality dimensions, the TIE would only have a modest relation to Agreeableness and Openness [38].

#### Trait EI (self-report)

Empirical research systematically confirms that self-reported scales do not predict ability assessments of EI well. Correlations between measures of trait EI and ability EI are invariably low [39] showing that the former belong to the realm of personality, whereas the latter pertain to the domain of cognitive ability. In direct tests, self-judgment-based responses are not highly correlated with measured abilities of perceiving, using, understanding, and managing emotions. Therefore, we expect significant albeit weak correlations of self-reported EI with the results of the TIE.

#### Perception of emotions

Convergence among the most widely used ability test of EI (MSCEIT) and ability measures of emotional perception is rather low. For example, correlations between the MSCEIT and the Japanese and Caucasian Brief Affect Recognition Test [40] are no higher than *r* = .18; with “facial blends” [41] reaching only *r* = .14, while those of the Reading the Mind in the Eyes Test [42] reach *r* = .56. The first studies using the TIE show significant relationships between its results and the ability measure of emotional perception. Wojciechowski and colleagues [43] found that all four TIE branch scores were significantly correlated with the results of a computer test measuring individual effectiveness in recognizing facial expressions (The Face Decoding Test, FDT). Analyses revealed a systematic pattern of positive relationships between the TIE and FDT, with Pearson’s *r* ranging between .20 and .38, all significant at *p*<.01. We expect similar results in this study.

#### Gender

Although studies based on self-reports bring disparate findings concerning gender differences in EI [11], [14], , when performance indicators of EI are used, clear and repetitive results are observable: women score higher than men, and these differences are sometimes as huge as one standard deviation [4], [9]. We expect similar gender differences (with females scoring higher) in the present study.

#### Age

According to the theory, EI should increase with age due to the accumulation of knowledge about emotion and its social context [46]. Nevertheless, studies designed to confirm this effect bring mixed results. Some researchers report significantly better scores on all four EI branches for older adults [7], some investigators [46] show older adults outperform younger participants on three out of four EI dimensions (no difference in perceiving emotions), while others – find no significant association between age and ability EI [47], [48] or even negative correlation between age and emotional perception [26], [49]. Most recently [50] it has been found that older people have lower scores than younger people for total EI and for perceiving, facilitating, and understanding emotions, whereas age is not associated with managing emotions.

Visibly, according to some studies, EI grows with time, but in agreement with the others it declines with age as any other cognitive ability. Paradoxically, it might not be a contradiction. Probably, from childhood, through adolescence, until middle and old age people develop emotional abilities and gather the experiences building their EI. Inevitable age-related cognitive decline happens only in very old age and does not equally affect all aspects of EI. It seems that older people have difficulty perceiving emotions [51], but may outperform younger people in managing emotions. Therefore, we expect small albeit significant age related gains in each EI branch until middle age, and a small decline for the subscales of perceiving, using and understanding emotions (but not managing) for the oldest participants. Thus, for the three branches a quadratic relationship with age is hypothesized, whereas for the fourth one we anticipate a systematic linear growth across the lifespan.

## Method

### Ethical Statement

The study was approved by the ethics committee of the Institute of Psychology, the Jagiellonian University in Krakow. Participants provided written informed consent. In the case of participants under 18, written informed consent was obtained both from the participant and the guardian.

### Participants

We analyzed data from 4642 people, combining about 30 studies conducted by our team and collaborators with broad experience in empirical research. Participants volunteered for studies with either no or little compensation (course credits or money). There were 2664 females and 1673 males in the sample (missing N = 305). The mean age of the sample was 25.47 (*SD* = 9.15, range 16–67). There were 644 high-school students, 1726 university students, and 773 employees in the sample (missing N = 1499). The structure of education in the sample of employees was as follows: primary N = 23, vocational N = 33, secondary N = 227, higher N = 247 (missing N = 243).

### Measures

#### Ability test of EI

Emotional intelligence was measured with the TIE. The test consists of four subtests representing consecutively *Perception*, *Understanding*, *Facilitation*, and *Management* of emotions (four labels of TIE subscales are slightly different from the labels of MSCEIT, but they pertain to the same theoretical model). Each subtest is defined by six item parcels. An item parcel consists of one problem situation in which people perceive, experience, manifest or use emotions, followed by three alternative responses. This is an example of such item parcel:

*Sophie hits the table with a fist. She frowns, her face is glowing, and her teeth are clenched. Most probably:*

a) *She is watching a popular show on TV*
*1 …… 2 …… 3 …… 4 …… 5*
b) *Once again she hurt her finger while cutting bread*
*1 …… 2 …… 3 …… 4 …… 5*
c) *She was just told by a colleague that he will not help her to prepare an important project, because he is leaving for a last-minute holiday*
*1 …… 2 …… 3 …… 4 …… 5.*

The three answers are related to the same emotional problem, however, each of them asks about the accuracy of a different strategy or perception, and therefore they can be treated as separate items. The whole test consists of two parts with different instructions. In the first part, referring to *Perception* and *Understanding*, participants are asked to reflect on feelings and thoughts of persons who were involved in the described situations. The task is to evaluate, on a 5-point Likert scale anchored at the ends with a “very bad answer” and a “very good answer,” the probability that a person involved in the situation experiences alternative emotions. In the second part, referring to *Facilitation* and *Management*, test-takers are asked to indicate the most advisable action that a protagonist should implement in order to solve the problem. The task is to judge, on a similar 5-point Likert scale, the level of appropriateness of each of the three actions described on the answer sheet, ranging from “very ineffective” to “very effective.” Scoring is based on the similarity of a testee’s responses with answers provided by the panel of experts (52 professionals, including 13 psychotherapists, 14 trainers of management, and 25 HR specialists). The points are summed up separately for all branches and for the whole test.

#### Self-reported EI

Self-reported EI was assessed via the SSEIT [18], a brief scale based on Salovey and Mayer’s [2] original definition of EI. Individuals are instructed to give their level of agreement to 33 statements describing different aspects of emotional life (e.g., “I know why my emotions change”) on a scale ranging from 1 (strongly agree) to 5 (strongly disagree). The original English version adapted to Polish [52] in this research reached an internal consistency of .91.

#### Perception of emotions

The ability of perceiving emotions in nonverbal signals was measured with the SIE-T, an instrument tapping the ability to recognize emotions in facial expressions [53]. The internal consistency was .84.

#### Fluid intelligence

Raven’s Advanced Progressive Matrices–RAPM [54], showing an internal consistency of .87, served as a tool to evaluate fluid intelligence.

#### Verbal intelligence

The test based on Horn and Cattell’s [55] theory of crystallized intelligence served as a tool for assessing verbal intelligence. The participants are asked to classify 120 words to one of the following categories: (1) art, (2) biology, (3) science, (4) literature, or (5) geography and history. The score is calculated as the number of correct answers given in five minutes.

#### Personality

To assess personality dimensions, we employed NEO-FFI [56], [57] showing the following internal consistencies (α): .77, .68, .82, .80, and .68 for Extraversion, Agreeableness, Conscientiousness, Neuroticism, and Openness to Experience, respectively.

### Procedure

Since the TIE is a paper-and-pencil test, it was possible to gather the data in small groups, normally not exceeding 10 persons. The participants worked in quiet settings, completing the test in a limited time of 30 minutes. Other instruments were administered according to standard instructions, usually after the participants completed the TIE test. The entire study lasted for 1 to 2 hours.

## Results

The main analyses (descriptive statistics, factor structure) were accomplished using the whole sample. For other analyses (convergent and discriminant validity), we took into account those participants who completed relevant tests or questionnaires (e.g., RAPM or NEO-FFI).

### Descriptive statistics

Table 1 provides basic descriptive statistics for four subscales and the total score of the TIE. The results are slightly negatively skewed, although neither skewness nor kurtosis indicators exceed an absolute value of 1.0, thus the score distributions may be treated as approximately normally distributed. Since neither floor nor ceiling effects can be discerned, it seems that the TIE provides enough space for assessment of individual differences in EI.

**Table 1:** Descriptive statistics.

	Perception	Understanding	Facilitation	Management	TIE total
Mean	7.75	7.15	6.82	6.30	28.03
SD	1.78	1.64	1.55	1.43	5.24
Range	9.85	9.94	10.34	9.27	30.52
Skewness	−.75	−.59	−.52	−.47	−.81
Kurtosis	.27	−.03	.03	−.23	.52

### Factor structure and intercorrelations

In order to verify competing structural models of the TIE, the results have been subjected to confirmatory factor analysis with the LISREL software and its pre-processor, PRELIS [58]. We investigated eight models, in groups of four. The first group was tested using an oblique-factor modeling strategy: the four-factor model, based on the underlying theory of EI, the one-factor model, assuming the existence of the general EI factor, and two versions of the two-factor solution. The first (Two-factor A, Table 2) corresponds to the area division by Mayer and Salovey [2]. The second version (Two-factor B, Table 2) corresponds to the results obtained in the pilot study [25] in which *Perception* and *Understanding* entered the first factor, whereas the dimensions of *Facilitation* and *Management* formed the second.

**Table 2:** Exact and close-fit statistics/indices (WLS) for TIE models.

Model	χ^2^ (*df*)	χ^2^/*df*	AIC	GFI	AGFI	RMSEA
Traditional oblique-factor modeling						
1. General factor	1143.40 (252)	4.54	1239.40	.981	.977	.028
2. Two-factor A	1136.81 (251)	4.53	1234.81	.980	.965	.035
3. Two-factor B	998.30 (251)	3.98	1096.30	.983	.980	.025
4. Four-factor	958.28 (246)	3.90	1066.28	.984	.979	.025
Nested factor modeling						
5. Two-factor A	784.76 (225)	3.49	934.76	.987	.982	.023
6. Two-factor B	706.57 (225)	3.14	856.57	.988	.984	.021
7. Three-factor	749.75 (228)	3.29	893.75	.987	.983	.022
8. Four-factor	717.54 (218)	3.29	881.54	.988	.983	.022

Note. N = 4642;

χ^2^/df–proportion of chi square to degrees of freedom [74].

GFI–Goodness of Fit Index [75].

AGFI–Adjusted Goodness of Fit Index [75].

RMSEA–Root Mean Square Error of Approximation [76].

AIC–Akaike Information Criterion (χ^2^/+2t).

Two factors A–Factor I: Perception and Facilitation, Factor II: Understanding and Management;

Two factors B–Factor I: Perception and Understanding, Factor II: Facilitation and Management.

The second group of models was analyzed using a nested factor modeling strategy [26]. In addition, in this case we tested a four-factor model and 2 two-factor solutions, as well as the novel model with three nested factors, endorsed by Palmer and colleagues. For the analyses we applied weighted least squares (WLS) estimation method. All solutions obtained an acceptable goodness of fit indicators (Table 2). Although the A and B two-factor models simply cannot be compared with each other due to an equal number of degrees of freedom, it seems obvious from all the applied fit indices that the solution proposed by the authors of the test (i.e., “B” model) is, in case of the TIE, definitely better than the original area division proposed by Mayer and Salovey [2]. The difference is visible both in traditional and nested factor modeling. For the oblique-factor group of models, the four-factor and two-factor “B” solutions revealed the lowest *χ^2^/df* ratio, i.e., proved to be the best fit-to-data. A comparison of the two models revealed supremacy of the four-factor model (*χ^2^_diff_* = 40.02, *df_diff_* = 5, *p*<.001) presented in Table 3.

**Table 3:** TIE standarized parameter estimates (WLS) for oblique-factor (Model 4) and nested factor (Models 6 and 8) models (N = 4642).

Item parcel	Model 4				Model 6			Model 8				
	P	U	F	M	‘g’	A1	A2	‘g’	P	U	F	M
p1	.41				.25	.24		.31	.19			
p2		.35			.03	.38		.25		.42		
p3		.49			.21	.36		.45		.28		
p4		.49			.23	.34		.44		.32		
p5	.54				.10	.53		.20	.52			
p6	.52				.26	.34		.31	.34			
p7	.49				.23	.33		.34	.26			
p8	.62				.29	.41		.41	.34			
p9	.56				.27	.37		.38	.30			
p10		.49			.35	.21		.49		.16		
p11		.49			.43	.14		.50		.10		
p12		.57			.43	.22		.56		.18		
p13			.51		.31		.29	.77			−.40	
p14				.31	.49		.14	.43				−.16
p15			.57		.30		.37	.95			−.62	
p16			.38		.35		.09	.48			−.13	
p17			.44		.17		.34	.79			−.58	
p18			.51		.40		.20	.65			−.17	
p19				.49	.25		.33	.39				.23
p20				.56	.16		.50	.35				.41
p21			.55		.41		.22	.71			−.22	
p22				.26	.09		.22	.18				.18
p23				.43	.06		.45	.21				.40
p24				.20	−.01		.26	.10				.21

Note. ‘g’–general EI factor, P–Perception, U–Understanding, F–Facilitation, M–Management, A1–Area 1 (Experiential), A2–Area 2 (Strategic).

The nested factor modeling strategy [26] resulted in a better fit to model in each of the analyzed cases, with all models significantly better fit in the nested version. Adding the “g” EI factor significantly improves the analyzed models’ fitness. Using the nested modeling strategy we have also confirmed the advantage of the four-factor solution over the two-factor “B” model (*χ^2^_diff_* = 49.85, *df_diff_* = 7, *p*<.001). These two models revealed the highest fit indices, surpassing the three nested factors solution that proved most appropriate in the case of MSCEIT (*χ^2^_diff_* = 43.18, *df_diff_* = 3, and *χ^2^_diff_* = 93.03, *df_diff_* = 10, respectively for two- and four-factor models; both significant at *p*<.001). Altogether, the CFA results suggest that the four-factor and two-factor “B” structures with nested factors should be preferred over the competing models.

Based on the goodness of fit indices reported above, we chose the three models to present their factor loadings (Table 3). Note that in model 8, the *Facilitation* factor loadings become negative, suggesting that the model may be overestimated. The case is similar to the nested two-factor model analyzed by Palmer and colleagues [26]. Albeit their model had an excellent fit, it was considered unacceptable. Therefore, model 8 must be treated with high caution, even if it seems adequate when analyzed using oblique modeling. It is interesting that exactly as in the Mayer–Salovey–Caruso Emotional Intelligence Test [16], *Facilitation* proved to be the “black sheep” of EI abilities. In all, the two-factor “B” model in its nested version seems the most reliable solution with respect to the factorial structure of the TIE.

The correlational analyses revealed that four subscales of the TIE are mutually intercorrelated and strongly related to the total score (Table 4).

**Table 4:** Correlations between TIE subscales and the total score.

	P	U	F	M	TIE total
Perception (P)		.63***	.55***	.50***	.84***
Understanding (U)			.54***	.50***	.83***
Facilitation (F)				.58***	.81***
Management (M)					.77***

Note: ***p<.0001.

### Reliability

The overall TIE reliability is *r* = .88. For the subscales, Cronbach’s alphas are: .70 (*Perception*), .69 (*Understanding*), .65 (*Facilitation*), .66 (*Management*). Additionally, we computed reliability indices for two parts of the test. For the first part (*Perception* and *Understanding*) Cronbach’s alpha is .81, and the respective value for the second part (*Facilitation* and *Management*), is .78. Coefficient alpha of the total score is adequate, reliabilities associated with the branch level scales are less satisfactory, although such estimates are common among tests in this area of research [41], [59], [60].

### Validity

We adopted several ways to evaluate the validity of the TIE (see Table 5). First, we looked at the discriminant validity, using two IQ tests. The total score of the TIE correlated with RAPM (N = 912) at the level of *r* = .35, *p*<.001, indicating a medium effect size [61] and with the G_c_ test (N = 474) at the level of *r* = .26, *p*<.001 (small effect size). The highest correlations with fluid and crystallized intelligence tests reached the subscale of *Understanding* (.37 and .36, respectively; medium effect size), which is consistent with the results reported by other researchers [60]. The remaining correlation coefficients ranged between .15 and .29. These medium strength intercorrelations suggest that EI, as measured with the TIE, and general intelligence are separate albeit interconnected mental abilities.

**Table 5:** Correlations between TIE scores and measures considered for the validity examination.

	RAPM (*N* = 912)	G_c_ test (*N* = 474)	NEO-FFI (*N* = 511)					SIE-T (N = 631)	SSEIT (N = 648)
			N	E	O	A	C		
TIE total	.35**	.26**	−.01	.03	.02	.16**	.01	.35**	.17**
*Branch-level scores*									
Perception	.29**	.15**	.01	.01	.04	.14**	−.01	.26**	.11**
Understanding	.37**	.36**	.07	−.01	.03	.11**	.05	.37**	.08
Facilitation	.26**	.20**	−.06	.07	−.01	.12**	.01	.27**	.18**
Management	.21**	.18**	−.05	.03	.01	.13**	.02	.27**	.12**

*p<.05,

**p<.01.

To evaluate the discriminant validity of the TIE we analyzed its relation to NEO-FFI. In our study (N = 511), only Agreeableness correlated with TIE’s subscales (coefficients ranged between .11 and .14, *p*<.01) and the total score (*r* = .16, *p*<.01), revealing a small effect size according to Cohen [61]. These correlations were weak, explaining about 2.5% of common variance, at the most. Thus, our test proved its independence of personality, similarly to other ability measures of EI based on performance criteria, such as MSCEIT [30].

Looking at the convergent validity of the TIE, we found that the total score of the TIE correlated with the SSEIT at a significant but low level (*r* = .16, *p*<.001, N = 648). As to the subscales, the correlation coefficient with SSEIT was insignificant for *Understanding*, while for others ranged from *r* = .11, *p*<.01 (*Perception*) to *r* = .18, *p*<.01 (*Facilitation*), thus only oscillating around the threshold of a small effect size [61]. The correlations between the TIE and the SIE-T were higher than with the SSEIT (*r* = .35, *p*<.001, N = 631), ranging from *r* = .26, *p*<.01 (*Perception*) to *r* = .37, *p*<.01 (*Understanding*) at the subscale level, showing medium effect sizes [61]. The self-report measure of EI revealed weaker relationships with the TIE than the ability test, despite the fact the latter referred to only one specific aspect of EI, namely: recognition of expressions of emotions.

The validity of an EI test can be evaluated not only via correlating its results with other instruments of the same kind, but also through analysis of group differences. In our study, women outperformed men in every subscale of the TIE, and consequently in the total score (Table 6). All differences are highly significant and the effect sizes are remarkable.

**Table 6:** Gender differences in TIE: subscales and total score (N = 4369).

	Males	Females	Difference	*t* (df = 4368)	*p*	*d*
Perception	7.40 (1.8)	8.03 (1.7)	.63	11.6	<.001	−.36
Understanding	6.78 (1.6)	7.43 (1.6)	.65	13.1	<.001	−.41
Facilitation	6.55 (1.6)	7.02 (1.5)	.47	9.8	<.001	−.30
Management	5.88 (1.4)	6.57 (1.4)	.69	15.8	<.001	−.49
TIE total	26.62 (5.2)	29.06 (5.0)	2.44	15.5	<.001	−.48

*Note.* Standard deviations are indicated in parentheses. d–Cohen’s effect size indicator.

In case of the TIE, the effect of age was also confirmed. The correlation coefficient between age and the general TIE score was *r* = .05, *p*<.001 (N = 4530). The effect was similar for three out of four of TIE branch scores, with Pearson’s *r*s of .04, *p*<.01 for *Perception*, .05, *p*<.01 for *Understanding*, and .06, *p*<.001 for *Management*. Only for *Facilitation* the correlation with age was not significant, *r* = .02, *p* = .12. These results are in line with conclusions formulated by Mayer, Caruso, and Salovey [7]. Although the effect is very small and does not reach the small effect size threshold proposed by Cohen [61], it does not differ from EI-age correlations obtained for MSCEIT [26]. We did not find any evidence supporting the formulated claims on curvilinear changes in EI branches across the lifespan.

## Discussion

The main aim of the present study was to test the psychometric properties and factor structure of the new ability test of EI. We assumed that TIE’s results would conform to the theoretical assumptions of the four-branch structure of EI, specified by Mayer and Salovey [22]. Most of the formulated hypotheses were confirmed.

The TIE proved its suitability as a reliable test of emotional intelligence. The internal consistency measures were rather high, as far as the total score is concerned, or mostly acceptable, if the particular subscales are taken into account. The internal consistency score at the level of .88 is a result very similar to the one usually found concerning Raven’s Matrices [54] or the MSCEIT [41], [62], [63], the most widely used tests of IQ and EI. Reliabilities associated with the subscales of the TIE were smaller, although such estimates are not unique among tests in this area of research. To point out only a few examples concerning MSCEIT: Caruso [64] reported a Cronbach’s of .74 (Understanding Emotions) and .76 (Managing Emotions), Roberts et al. [60] revealed .67 (Using Emotions to Facilitate Thinking), .68 (Understanding Emotions), and .68 (Managing Emotions). More recently, Austin [41] showed .58 (Using Emotions to Facilitate Thinking), .66 (Understanding Emotions), and .66 (Managing Emotions); and finally, Lopes and colleagues in one of their studies [65] obtained for Managing Emotions a split-half reliability of .57. Because of the fact that reliability measures concerning subscales of the TIE do not differ substantially from other tools of this kind [66], both the total and subscales may be used in the research, while for individual diagnosis it seems advisable to rely on the total score.

The results of the TIE conform to the theoretical assumptions about the nature and structure of EI, specified by Mayer and Salovey [2], [7], [22]. This finding is good both for the theory, as its empirical support, and for the test, as an argument for its theoretical validity. At the same time, our study suggests that the between-branch connections may be conceptualized in a different manner than in the original theory. In Mayer and colleagues’ original conceptualization, perceiving and using emotions formed the experiential factor, whereas understanding and managing emotions loaded the factor of strategy [16]. This division is based on an observation that some emotional abilities enhance emotional knowledge and make one “closer” to one’s own and others’ emotions, and therefore comprise the “hotter” part of EI, whereas other abilities are usually treated as useful tools for “cold,” strategic regulation of emotion. However, this division of abilities remains a problematic issue. Whereas perception is clearly experiential, and management is definitely strategic, the understanding and facilitation branches are much more ambiguous. The former is manifested mainly in adequate labeling of emotions, as well as linking them with their causes and consequences. Accurate labeling is necessary for differentiation of similar emotional states; thus, to distinguish two different albeit similar affective states one needs to accurately feel these states, which makes the understanding branch experiential, at least to some degree. On the other hand, facilitation, also labeled “using emotion to facilitate thought” [2], [7], has much in common with processes of meta-cognitive control, and thus remains a very “applicable” and therefore strategic dimension.

Our results imply that the *Perception* branch may be in fact situated closer to *Understanding* than *Facilitation*, while the latter seems to be more strongly connected to *Management* than to *Understanding*. This finding can lead to modification of Salovey and Mayer’s theory. Nevertheless, we suggest this interpretation with caution, realizing that our outcomes may result either from specific operationalization of respective branches of EI or from cultural specificity. However, our findings are generally consistent with the underlying theoretical assumptions and empirical data concerning the structure of emotional intelligence as a set of four abilities.

Confirmatory Factor Analyses revealed that although four-factor models were characterized with the best fit indices, they should be treated with caution due to unexpected negative loadings of Facilitation branch. Therefore, the two factor solution with general-EI loading on each area seems much less ambiguous, and, in the light of the presented data, should be preferred over the remaining ones. The supremacy of the nested models suggests that EI, as measured with the TIE, has much more to do with intelligence (in the way in which Charles Spearman would want to see it, as loaded with general and specific factors), than with personality. Thereby, we provide further evidence for “the intelligence of EI” [7], based not only on the test’s convergent/divergent validity, but also on its factorial structure.

Many studies introducing new EI methods face the problem of weak convergent validity. Our results seem to be in line with the empirical data published to date. The total score of the TIE correlated with self-reported EI at a significant but low level oscillating around the threshold of a small effect size [61]. Taking into account that both tools are based on the same theoretical model, one might expect much higher correlations, although such result is not an exception. The most elegant example was provided by Brackett and colleagues [9], who developed a self-judgment scale based on the Four-Branch Model and found a correlation of only *r* = 0.19 with the MSCEIT. More commonly used self-judgment scales of EI, such as Bar-On’s EQ-i [14] or the SSEIS [18] predict MSCEIT results better, but still at a pretty low level [12], [67]. MacCann and Roberts’s Situational Test of Emotional Understanding and Situational Test of Emotional Management [68] correlated with MSCEIT of .33 and .36, respectively. A very similar correlation of .36 has been found between the TIE and the PKIE, a self-reported measure of EI [69]. The problem of very low correlations between different measures of EI undoubtedly stems from inconsistences on a theoretical and psychometric level, and leads to the conclusion that self-assessed EI and ability EI measured by performance tests are different constructs.

Despite the disparate forms of measurement methods, the correlations of the TIE and an instrument tapping the ability to recognize emotions in facial expressions were higher, revealing medium effect sizes [61]. As significant relations cannot be assigned to shared method variance in this case, such result provides support for the construct’s convergent validity.

Compared with the convergent validity evidence, the discriminant validity evidence for the TIE is promising. According to the ability-based approach, emotional intelligence is not a personality trait, and thus its measures should not share much variance with major personality dimensions. Many empirical studies [9], [12], showed that EI shares only a small fraction of common variance with personality. In line with that, the TIE proved its independence of personality. Weak, although significant, correlations with Agreeableness, and the lack of any other relationships with the remaining Big Five dimensions, should be interpreted as evidence that the TIE does not cover preferences, habits, or inclinations. The very moderate correlations between the TIE and measures of general intelligence suggest that EI is a set of mental abilities, related to intelligence, but quite independent of it. These findings are also consistent with EI literature [32], [60], [66], [70].

Most tests of ability EI reveal systematic gender differences. Research proves that women tend to show greater knowledge of emotional experiences, provide more complex descriptions about emotions, and use a broader emotional vocabulary [71]–[73], and thus consequently regularly score higher than men on EI ability tests [4], [9]. Also in our study, women outperformed men in every single subscale of TIE, and consequently in the total score (with remarkable effect sizes). Such confirmation of the generally recognized phenomenon of women’s advantage concerning emotional abilities is additional evidence supporting the new test’s validity.

According to Mayer and Salovey’s theory, EI should increase with age due to the accumulation of knowledge about emotion and its social context [7], [46]. Our results are in line with such prediction. Although the effect is small, it does not differ from EI-age correlations obtained for MSCEIT [26]. Since our sample did not include participants older than 70, we did not discover age-related cognitive decline in EI.

## Conclusions

Although it is impossible to provide exhaustive psychometric evidence for a new measure in one study, the results of the present investigation are encouraging. In general, our findings are consistent with prior studies on EI ability measures. The TIE meets psychometric standards concerning reliability, theoretical validity (factorial structure), and discriminant validity. Undeniably, the results for convergent validity seem less satisfactory, although such problem is widespread in this domain of research. Convergent validity of EI measures remains a controversial topic and continually elicits lively debate among EI researchers. A significant limitation of our study is the fact that we did not use the MSCEIT to prove convergence between two performance tests. Definitely, a high correlation between the MSCEIT and the TIE would be adequate evidence for the convergent validity of the latter. Unfortunately, the MSCEIT is not available in Poland, and hence such analyses were unattainable.

The present empirical investigation involved more than 4000 participants from different populations and applied the most widely used measures of human personality and intelligence. Nevertheless, it only provides the groundwork for subsequent systematic research. The evidence for the validity of the TIE will probably arise from multiple studies with diverse samples and a variety of theoretically related criteria. In future studies, the TIE should be validated against other measures of emotional abilities and indicators of predictive validity which hopefully show how individuals with higher (and lower) EI handle situations in which emotions play an important role.

Further progress in the measurement of EI is undoubtedly required. In our opinion, future studies should provide an in-depth look into the true nature of “general” EI. Usually, EI is treated as a simple sum of the four branch scores, reflecting the four elementary groups of emotional abilities. However, the supremacy of nested models revealed in the present paper, suggest that the issue is more complex. It appears that a general, unspecific mental-emotional capacity does exist. It loads each of the particular branches, but cannot be reduced to their sum. This conclusion could be a starting point for a revision of the established ability EI model, so that this set of emotional skills “behaves” in a way consistent with how Spearman described “traditional” intelligence, with each performance loaded with both a general factor and specific factor(s).

The TIE has been developed as an alternative ability measure of emotional intelligence. Our aim was to create a valid and reliable instrument to tap the multidimensional construct of EI, which does not require considerable time to administer, and is easy to use for scientific and practical purposes. We believe that this goal has been accomplished. The TIE is useful and valuable because it incorporates a distinctive set of characteristics. Most of the existing EI measures present only one or two of those qualities, while the TIE has it all. First, it displays the advantages of the performance tests by measuring actual abilities of emotional perception and reasoning, not motivations, personality, or self-esteem. In comparison to other ability scales, it covers the whole spectrum of emotional abilities, fully reflecting the theoretical model of EI. In comparison to the MSCEIT, finally, which also meets the above criteria, TIE is shorter, easier to administer and based on a different cultural context. That being the case, we hope the TIE will enrich the collection of available EI tests and will serve to advance the domain.
